# Management and outcomes of real-world use of non-vitamin-K oral anticoagulants (NOACs) in patients with atrial fibrillation: experience of a dedicated NOAC clinic

**DOI:** 10.1007/s12471-019-01330-y

**Published:** 2019-11-26

**Authors:** A. J. W. M. de Veer, N. Bennaghmouch, M. C. E. F. Wijffels, J. M. ten Berg

**Affiliations:** Department of Cardiology, St. Antonius Hospital, Nieuwegein, The Netherlands

**Keywords:** Anticoagulation, Atrial fibrillation, Non-vitamin‑K oral anticoagulants

## Abstract

**Background:**

Current guidelines recommend non-vitamin‑K oral anticoagulants (NOACs) as the first-choice therapy for stroke prevention in patients with atrial fibrillation (AF). The use of drugs in a clinical trial setting differs from that in real-world populations. Real-world data are important to accrue more heterogeneous patient populations with respect to co-morbidities and co-medication use. The aim of this study was to evaluate the use of NOACs in daily practice in a large tertiary hospital in the Netherlands.

**Methods:**

A single-centre prospective study was conducted among all patients with AF using a NOAC in the St. Antonius Hospital between 2013 and June 2017. The outcomes were the rates of any bleeding, stroke/transient ischaemic attack, mortality, discontinuation rate and adverse drug reactions.

**Results:**

In total, 799 patients were enrolled with a mean follow-up of 1.7 years. Mean age was 69.8 (SD ± 11) and 61.2% were male. Mean CHA_2_DS_2_-VASc score was 2.8 (SD ± 1.6) and mean HAS-BLED score was 1.4 (SD ± 0.9). Bleeding occurred in 6.0, major bleeding in 1.8, stroke in 1.2 patients per 100 patient-years, and 87 patients (10.9%) died during the follow-up period. Adverse drug reactions were reported by 59 patients (7.4%). Finally, 249 patients (31.2%) reported a temporary interruption and 132 (16.5%) permanent discontinuation of NOAC treatment, of whom 33 (25%) patients switched to a vitamin‑K antagonist.

**Conclusions:**

We observed low rates of bleeding and adverse drug reactions. However, rates of mortality and discontinuation were relatively high. These results could possibly be explained by the real-world nature of the data including higher-risk patients.

**Electronic supplementary material:**

The online version of this article (10.1007/s12471-019-01330-y) contains supplementary material, which is available to authorized users.

## What’s new?

- This is the first report on real-world use of all four non-vitamin‑K oral anticoagulants (NOACs) in atrial fibrillation (AF) patients in a large cardiology centre in the Netherlands.
- We found rates of stroke and bleeding that were similar to those of phase III randomised controlled trials (RCTs) of NOACs in AF patients.
- Significantly, all-cause mortality was higher and discontinuation rates were lower than in these RCTs.
- This study confirms that the use of NOACs in everyday clinical practice is safe and efficacious.

## Introduction

Current guidelines recommend non-vitamin‑K oral anticoagulants (NOACs) as the first-choice therapy for stroke prevention in patients with atrial fibrillation (AF) [1–4]. The currently available NOACs have proved to be either comparable (dabigatran 110 mg, rivaroxaban and edoxaban) or superior (dabigatran 150 mg and apixaban) to warfarin in preventing stroke or systemic embolism and have demonstrated either a similar (dabigatran 150 mg and rivaroxaban) or superior (dabigatran 110 mg, apixaban and edoxaban) safety profile, with reductions in major and minor bleeding rates [5–9]. All four agents have been associated with a statistically significant reduction in the rates of intracranial haemorrhage compared with warfarin. Despite these promising results, with the introduction of the NOACs in the Netherlands for stroke prevention in AF patients, questions were raised concerning the safety, efficacy and cost-effectiveness of these new drugs in daily practice [10, 11]. The main argument was that the use of drugs in a clinical trial setting differs from that in real-world populations, as randomised controlled trials generally have stricter inclusion criteria and structured monitoring schemes with a shorter follow-up interval. When evaluating the safety and efficacy outcome events associated with NOACs, real-world data are important to accrue more heterogeneous patient populations with respect to co-morbidities and co-medication use. Therefore, the questions regarding the safety and efficacy of NOACs in patients with AF treated in real-world clinical settings in the Netherlands will remain until real-world data are presented. As required by the Dutch government, we referred all patients to our tertiary NOAC clinic with the purpose of monitoring the safety and efficacy of the introduction of NOACs.

The objectives of this study were to describe demographics, to evaluate the rates of bleeding, mortality and thromboembolic events, to evaluate the rates of reported adverse drug events, and to evaluate treatment persistence in patients with AF treated with NOACs in daily practice in a large tertiary hospital in the Netherlands with a tertiary NOAC clinic.

## Methods

The St. Antonius Hospital NOAC registry is an observational, single-centre, prospective study in patients with AF treated with a NOAC for stroke prevention.

Because of the non-interventional character of the study the Ethical Committee (EC) confirmed that the Medical Research Involving Human Subjects Act (WMO) does not apply to this study and therefore official ethical approval of this study by the EC is not required. Declaration approval of the Institutional Board Review (LMTE) was obtained. A local study protocol is available. The study was conducted in accordance with the Declaration of Helsinki.

The NOAC clinic was initiated in 2013 with the purpose of monitoring the introduction of NOACs in our centre. All patients visited the outpatient clinic, where they consulted a physician with extensive knowledge about all NOACs and clinical trials. The patients were referred by either a cardiologist, neurologist, internal medicine doctor, or a general practitioner. During the consultation, the patient received information regarding AF and anticoagulation in general. Furthermore, patients received tailored education on and explanation about the use of a NOAC, the (modifiable) bleeding risks and the required laboratory assessments. Risk factors for bleeding were checked and reasons to reduce the NOAC dose were assessed. Also, all patients received an anticoagulation card [3]. This card contains information regarding the anticoagulation therapy, as well as other drugs patients are using. Every patient received a direct telephone number and/or e‑mail address of a dedicated physician in order that that physician could be easily reached by a patient. When needed, a follow-up visit at the NOAC clinic was planned.

Follow-up was done through the regular outpatient clinic of the cardiology department, or by the general practitioner, whichever was appropriate. The general practitioner and/or referring physician received documentation about the initiation of NOAC therapy.

### Study population and follow-up

We included all consecutive patients who visited or consulted the NOAC clinic. Because of the observational design, no exclusion criteria apply. Patients could be either (N)OAC naïve or (N)OAC experienced. Decisions with regard to NOAC choice, continuation or changes were at the discretion of the treating physician.

Baseline characteristics at the NOAC start date were collected. Follow-up data were collected by reviewing electronic patient files and follow-up was performed from the start of NOAC treatment until the last (outpatient) visit in our hospital or until a patient died, with a final follow-up date of 24 May 2018. No study-specific visits were performed. We did not assess medication adherence. Mortality was checked at the Dutch civil registry.

### Study outcomes

The primary outcome was the occurrence of any in-hospital bleeding or bleeding reported at the outpatient NOAC clinic or regular outpatient cardiology clinic during long-term follow-up. The co-primary outcomes were the rates of stroke/transient ischaemic attack (TIA) and mortality at long-term follow-up. Secondary outcomes were the rates of pulmonary embolism, deep venous thrombosis, myocardial infarction, NOAC therapy discontinuation and adverse drug reactions. Furthermore, we collected the reasons for NOAC therapy discontinuation; whether and to which drug patients switched; the number of patients who changed to another type or dose of NOAC. The predefined definitions of endpoints are listed in the Electronic Supplementary Material (Appendix 1).

Additionally, we performed an on-treatment event analysis. In all patients who permanently discontinued their NOAC therapy, follow-up was censored 3 days after discontinuation. Thus, only events that occurred during or within 3 days after discontinuation of treatment were evaluated.

### Statistical analysis

Descriptive analysis of the data was performed using summary statistics for categorical and quantitative (continuous) data. Continuous data are reported as means with standard deviation or medians with interquartile range. Categorical data are expressed as percentages. Distributions of categorical data were examined by the *Χ*^2^-test or Fisher’s exact test, as appropriate. Continuous data were compared using Student’s *t*-test or the Mann-Whitney U test, as appropriate. The analyses were performed using SPSS software for Windows, version 24 (IBM Corporation, Armonk, NY, USA).

## Results

### Patient characteristics

**Fig. 1:**
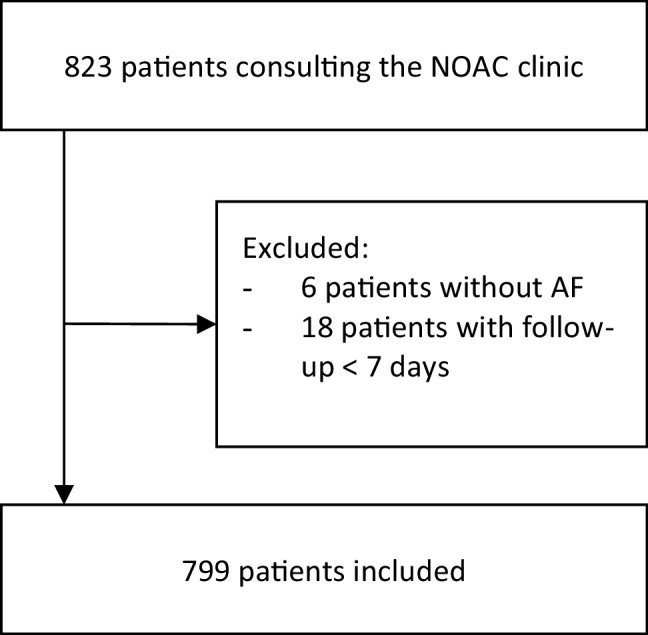
Flowchart of patient inclusion criteria. *NOAC* non-vitamin‑K oral anticoagulant, *AF* atrial fibrillation

Between 2013 and May 2017, out of 823 consecutive patients consulting the NOAC clinic, a total of 799 were included in the NOAC registry. Thus, 24 patients were not included because they did not start the NOAC therapy, or did not suffer from AF. A flowchart of the inclusion criteria is presented in Fig. 1. The mean follow-up was 1.7 ± 1.0 years. The proportion of male patients was 61.2%, and the mean age was 69.8 ± 11.0 years. Almost half of all included patients were antithrombotic therapy naïve (*n* = 350, 43.8%), 22.2% (*n* = 177) used a vitamin‑K antagonist and 29.3% (*n* = 233) used acetylsalicylic acid (ASA) at baseline. In all patients, ASA was discontinued after initiation of NOAC therapy, since in our centre we use NOAC monotherapy in patients with AF and coronary or peripheral artery disease. The mean CHA_2_DS_2_-VASc score was 2.80 ± 1.6 and the mean HAS-BLED score was 1.36 ± 0.9. All baseline characteristics are presented in Tab. 1. Baseline characteristics according to the adopted NOAC dose are presented in the Electronic Supplementary Material (Appendix 4).

**Table 1:** Baseline characteristics

Total number of patients included		799
Male gender, *n* (%)		489 (61.2)
Age, mean ± SD		69.8 ± 11.0
Age groups, *n* (%)	<65	228 (28.5)
	65–74	303 (37.9)
	≥75	268 (33.5)
BMI (kg/m^2^), mean ± SD		27.4 ± 5.0
CrCl (ml/min), mean ± SD		65.3 ± 13.7
	15–29, *n* (%)	5 (0.6)
	30–49, *n* (%)	71 (8.9)
CHA_2_DS_2_-VASc score^a^, mean ± SD		2.8 ± 1.6
CHADS_2_ score, mean ± SD		1.5 ± 1.2
HAS-BLED score^b^, mean ± SD		1.4 ± 0.9
Atrial fibrillation type, *n* (%)	De novo	356 (44.6)
	Paroxysmal	344 (43.1)
	Persistent	43 (5.4)
	Permanent	19 (2.4)
	Unknown	37 (4.6)
Prior stroke/TIA, *n* (%)		113 (14.1)
Prior PE/DVT, *n* (%)		30 (3.8)
Myocardial infarction, *n* (%)		102 (12.8)
Coronary artery disease, *n* (%)		170 (21.3)
Peripheral artery disease, *n* (%)		44 (5.5)
Congestive heart failure, *n* (%)		108 (13.5)
LVEF, *n* (%)	Normal (>50%)	473 (59.2)
	Reduced (<50%)	101 (12.6)
	Unknown	225 (28.2)
Hypertension, *n* (%)		455 (56.9)
Diabetes mellitus, *n* (%)		123 (15.4)
Current smoking, *n* (%)^c^		89 (11.1)
Alcohol abuse, *n* (%)^d^		127 (15.9)
Prior major bleeding, *n* (%)		23 (2.9)
Malignancy, *n* (%)		117 (14.6)
Prior use of antithrombotic therapy, *n* (%)	No	350 (43.8)
	ASA	233 (29.2)
	P2Y12‑i	60 (7.5)
	VKA	177 (22.2)
	LMWH	23 (2.9)
	Dipyridamole	10 (1.3)
NOAC type, *n* (%)	Dabigatran 150 mg	43 (5.4)
	Dabigatran 110 mg	15 (1.9)
	Rivaroxaban 20 mg	442 (55.3)
	Rivaroxaban 15 mg	65 (8.2)
	Apixaban 5 mg	163 (20.4)
	Apixaban 2.5 mg	19 (2.4)
	Edoxaban 60 mg	42 (5.3)
	Edoxaban 30 mg	10 (1.3)
Reason to initiate NOAC, *n* (%)	Labile INR on VKA	50 (6.3)
	Insufficient stroke prevention^e^	244 (30.5)
	Usability compared to VKA	92 (11.5)
	Bleeding with current therapy	6 (0.8)
	Adverse events	8 (1.0)
	Other	50 (6.3)
	AF de novo	349 (43.7)

*BMI* indicates body mass index, *CrCl* creatinine clearance calculated using the Cockcroft-Gault formula, *TIA* transient ischaemic attack, *PE* pulmonary embolism, *DVT* deep venous thrombosis, *LVEF* left ventricular ejection fraction, *ASA* acetylsalicylic acid,* P2Y12‑i* P2Y12 inhibitor, *VKA* vitamin‑K antagonist, *LMWH* low-molecular-weight heparin, *NOAC* non-vitamin‑K oral anticoagulant

^a^The CHA_2_DS_2_-VASc score reflects the risk of stroke, with values ranging from 0 to 9 and higher scores indicating greater risk

^b^The HAS-BLED score reflects the risk of major bleeding in patients with atrial fibrillation (*AF*) who are receiving anticoagulant therapy, with values ranging from 0 to 9 and with higher scores indicating greater risk

^c^Data missing on 102 patients

^d^Data missing on 204 patients

^e^For example, were on single/double antiplatelet therapy

The prescribed NOAC type and dose are shown in Tab. 1. Rivaroxaban was prescribed in 507 patients (63.4%), dabigatran in 58 patients (7.3%), apixaban in 182 patients (22.8%) and edoxaban in 52 patients (6.6%). The majority of the patients (86.4%) were prescribed the standard dose of NOAC. The most frequent reasons to initiate a NOAC were newly detected AF (*n* = 349, 43.7%) and insufficient stroke prevention (e.g. using single or double antiplatelet therapy).

### Outcome events

Bleeding occurred in 82 patients (10.2%), major bleeding in 25 patients (3.1%) and stroke/TIA in 24 patients (3.0%). This translates to a rate of any bleeding of 6.0 per 100 patient-years, a rate of stroke of 1.2 per 100 patient-years and a rate of TIA of 0.6 per 100 patient-years. Furthermore, 87 patients (10.9%) died during follow-up, which translates to a mortality rate of 6.4 per 100 patient-years. A total of 59 patients (7.4%) experienced an adverse drug reaction, with headache, pruritus and dyspepsia being the most frequently reported adverse drug reactions. All primary and secondary outcomes are listed in Tab. 2.

**Table 2:** Study endpoints

	Incidence	Incidence rate, events per 100 patient-years
*Stroke, n (%)*^*a*^	16 (2.0)	1.2
*TIA, n (%)*^*a*^	8 (1.0)	0.6
*Pulmonary embolism, n (%)*^*a*^	2 (0.3)	0.1
*DVT, n (%)*^*a*^	3 (0.4)	0.2
*Myocardial infarction, n (%)*^*a*^	12 (1.5)	0.9
*Bleeding, n (%)*^*a*^	82 (10.2)	6.0
– major bleeding^b^	25	1.8
– CRNM bleeding^b^	42	3.1
– minor bleeding^b^	32	2.4
*All-cause mortality, n (%)*	87 (10.9)	6.4
– CV death	44	
– non-CV death	43	
*Adverse drug reactions, n (%)*	59 (7.4)	
– fatigue, *n* (%)	2	
– skin rash, *n* (%)	2	
– dyspepsia, *n* (%)	7	
– intestinal complaints	2	
– malaise	3	
– pruritus	9	
– headache, *n* (%)	12	
– dizziness, *n* (%)	6	
– haematoma, *n* (%)	7	
– other, *n* (%)^c^	9	

*TIA* indicates transient ischaemic attack, *DVT* deep venous thrombosis, *CRNM bleeding* clinically relevant non-major bleeding, *CV death* cardiovascular death

^a^Only first event

^b^Total number of bleedings

^c^Psychological complaints, liver function, muscle ache, joint ache

When censoring patients 3 days after they discontinued the NOAC therapy, mainly the mortality rate was lower. All on-treatment event rates are presented in the Electronic Supplementary Material (Appendix 2).

The most frequent bleeding sites were gastrointestinal and genitourinary locations. All bleeding sites and their frequencies are presented in the Electronic Supplementary Material (Appendix 3).

### Discontinuation of NOAC therapy

Out of the 799 patients included, 132 (16.5%) permanently discontinued the NOAC therapy. The most important reason for this was the end of treatment according to a physician, e.g. a patient with a CHA_2_DS_2_-VASc score of 0 who underwent successful elective electrocardioversion and stopped the NOAC therapy a few weeks after. Other reasons were premature discontinuation because of adverse drug reactions, bleeding and fear of bleeding. Of these patients, 33 switched to a vitamin‑K antagonist and 71 did not continue on any OAC. Furthermore, a total of 249 patients (31.2%) reported a temporary interruption of NOAC treatment. All data regarding discontinuation of NOAC therapy are listed in Tab. 3. Notably, only one patient discontinued NOAC therapy because of renal failure.

**Table 3:** Permanent and temporary discontinuation of NOAC therapy

*Temporary discontinuation, n (%)*		249 (31.2)
*Permanent discontinuation, n (%)*		132 (16.5)
* Reason for discontinuation, n (%)*^a^	End of treatment	61
	Adverse drug reaction	26
	Thrombotic event	2
	Bleeding	19
	Fear of bleeding	10
	Interaction other drugs	3
	Renal failure	1
	Unknown	8
	Other	20
* Switch to, n (%)*	ASA	18
	VKA	33
	LMWH	6
	Other	1
	No antithrombotic therapy	71
	Unknown	3
*NOAC type change, n (%)*^b^		47 (5.9)
*NOAC dose change, n (%)*		46 (5.8)

*NOAC* non-vitamin‑K oral anticoagulant, *ASA* acetylsalicylic acid, *VKA* vitamin‑K antagonist, *LMWH* low-molecular-weight heparin

^a^More than one reason was possible

^b^Switch to another NOAC

## Discussion

In this real-world registry of AF patients treated with NOACs we provide a complete description of all patients that visited the NOAC clinic in a high-volume centre in the Netherlands. We also provide the adverse event rates and the treatment details.

This tertiary NOAC clinic was launched with the purpose of monitoring the safety and efficacy of the use of the recently introduced anticoagulation drugs. We extensively provided support and education to every patient starting a NOAC and coordinated their integrated care. We aimed to waive the concerns of non-adherence by comprehensive education and intense monitoring.

We compared our data with available data from the four pivotal randomised control trials (RCTs) of NOACs in AF: RE-LY, ROCKET-AF, ARISTOTLE and ENGAGE AF-TIMI 48 [5–8]. Mean age and proportion of male patients were similar. The baseline stroke risk of patients in this NOAC registry is somewhat lower than that of pivotal NOAC randomised trials with a mean CHADS_2_ score ranging from 2.1 to 3.5. The number of patients having experienced myocardial infarction was comparable. On the other hand, in our cohort the numbers of patients with a history of prior stroke, diabetes mellitus, hypertension and heart failure are noticeably lower.

In our registry, the any bleeding rate per 100 patient-years was 6.0 for the total population. This rate was found to be lower than in the pivotal randomised trials and available real-world data. The Apixaban for Reduction in Stroke and Other Thromboembolic Events in Atrial Fibrillation (ARISTOTLE) study found an annual incidence of any bleeding of 18.1% for apixaban [7]. Furthermore, the Effective Anticoagulation with Factor Xa Next Generation in Atrial Fibrillation—Thrombolysis in Myocardial Infarction 48 (ENGAGE AF-TIMI 48) trial found an annual incidence of any overt bleeding of 14.15% for high-dose edoxaban [8]. The Rivaroxaban Once Daily Oral Direct Factor Xa Inhibition Compared with Vitamin K Antagonism for Prevention of Stroke and Embolism Trial in Atrial Fibrillation (ROCKET-AF) and Randomised Evaluation of Long-Term Anticoagulant Therapy (RE-LY) trials did not provide incidences of any bleeding [5, 6]. However, the rate of major bleeding per 100 patient-years in the present registry was 1.8, and this rate is in the range of the phase III trial data. Namely, RE-LY reports annual incidences of 2.71% and 3.11% for dabigatran 110 mg b.i. d. and dabigatran 150 mg b.i. d. respectively, ARISTOTLE reports an annual incidence of 0.96%, ENGAGE an annual incidence of 2.75% and ROCKET-AF 3.6 major bleedings per 100 patient-years [5–8]. Major bleeding rates of 2.1–3.0 per 100 patient-years in patients using rivaroxaban and 2.8 per 100 patient-years in patients using apixaban were reported in the Dresden NOAC registry and the Xarelto for Prevention of Stroke in Patients with Atrial Fibrillation (XANTUS) registry [12–15]. A possible explanation for the relatively low any bleeding rate in our study might be the observational character and the absence of obligatory study-specific visits. This could have caused underreporting of any bleeding, mainly minor bleeding. Furthermore, in our cohort ASA was discontinued in all patients after initiation of NOAC therapy as opposed to the pivotal NOAC trials where this was left to the discretion of the physician. This difference might be another explanation for the lower any bleeding rate in our study.

The presented rate of stroke in our registry is consistent with the data of the pivotal NOAC trials. We report an incidence of 1.2 strokes per 100 patient-years, whereas the RCTs report rates ranging from 0.92% to 1.49% per year. Conversely, all-cause mortality in our registry was 6.4 patients per 100 patient-years, whereas the RCTs report all-cause mortality ranging from 1.9% to 4.0% per year. The XANTUS registry reports incidences of 0.7 strokes and 1.9 deaths per 100 patient-years, respectively. In the Dresden NOAC registry incidence of stroke and mortality is not reported. Korenstra et al. reported a rate of 0.6 strokes per 100 patient-years and a mortality rate of 2.0% per year [16].

Drug persistence is a major concern in stroke prevention because anticoagulant discontinuation potentially leaves patients unprotected against the risk of stroke. In our study, we showed that 16.5% of patients permanently discontinued their NOAC therapy, 8.8% being cases of premature discontinuation. In the pivotal NOAC trials, permanent discontinuation ranged between 21% and 25% with a mean follow-up of 1.8–2 years. Furthermore, in the Dresden NOAC registry, 223 patients (18.5%) permanently discontinued rivaroxaban during follow-up (554 days) [17]. These figures are in accordance with our results. Unfortunately, we did not register adherence to the prescribed NOAC therapy.

Our study is subject to limitations. First, some events may have been underestimated. Because of the observational nature of the study, some thrombotic and bleeding events might not be reported. In contrast, the mortality data were not underestimated, since mortality was checked at the Dutch civil registry. Another limitation is that we did not study medication adherence in this cohort. Also, there might be selection bias and confounding in this study because of its observational nature, as always with real-world evidence.

One of the strengths of this study is the completeness of the data. Since this registry is not a claim registry, we did not depend on the completeness and quality of the diagnosis coding in a claim database. For example, information on whether the proper NOAC dose was prescribed based on clinical and laboratory data is not frequently recorded in claims databases. Finally, we included patients who were using all four available NOACs. Other available registries in the Netherlands only reported on the use of a single NOAC [16, 18].

In conclusion, in this real-world registry of AF patients using NOACs, we showed that NOACs are safe and efficacious. The observed rates of major bleeding and stroke were similar, the all-cause mortality was higher, and the any bleeding and discontinuation rates were lower than those in the randomised trials. This study, including a real-world cohort of consecutive patients visiting a tertiary NOAC clinic, confirms the results of the RCTs in everyday clinical practice.

## Caption Electronic Supplementary Material

Appendix 1 – Definitions of outcomes. Appendix 2 – Events on-treatment analysis. Appendix 3 – Bleeding sites. Appendix 4 – Baseline characteristics according to dose group
