# Supplementary material for: Management and outcomes of real-world use of non-vitamin-K oral anticoagulants (NOACs) in patients with atrial fibrillation: experience of a dedicated NOAC clinic
Source: Neth Heart J. 2019 Nov 26;27(12):605–12. doi: 10.1007/s12471-019-01330-y (PMC6890914; doi:10.1007/s12471-019-01330-y)
Supplement: Supplementary file 1 — Appendix 1 – Definitions of outcomes. Appendix 2 – Events on-treatment analysis. Appendix 3 – Bleeding sites. Appendix 4 – Baseline characteristics according to dose group [file 12471_2019_1330_MOESM1_ESM.docx]

**Electronic supplementary material**

Appendix 1 – Definitions of outcomes

Appendix 2 – Events on-treatment analysis

Appendix 3 – Bleeding sites

Appendix 4 – Baseline characteristics according to dose group

1 – Definitions of outcomes

**1) ISTH bleeding**

*Major bleeding:*

This is defined as overt bleeding associated with:

- a fall in hemoglobin of ≥2 g/dL
- a transfusion of two or more units of packed red blood cells or whole blood
- occurrence at a critical site (intracranial, intraspinal, intraocular, pericardial, intra-articular, intramuscular with compartment syndrome, retroperitoneal)
- death.

*Clinically relevant, non-major (CRNM) bleeding:*

A clinically relevant minor bleed is an acute or subacute clinically overt bleed that does not meet the criteria for a major bleed but prompts a clinical response, in that it leads to at least one of the following:

- A hospital admission for bleeding, or
- A physician guided medical or surgical treatment for bleeding, or
- A change in antithrombotic therapy (including interruption or discontinuation of study drug).

*Minor bleeding:*

All other overt bleeding episodes not meeting the criteria for major or clinically relevant nonmajor bleeding.

**2) Stroke**

Stroke is defined as a new, sudden, focal neurologic deficit resulting from a presumed cerebrovascular cause that is not reversible within 24 hours and not due to a readily identifiable cause, such as a tumor or seizure.

Whenever possible, computed tomography scanning or magnetic resonance imaging should be used to assist in the classification. Stroke will be classified as:

- *primary hemorrhagic* – stroke with focal collections of intracerebral blood; hemorrhagic stroke will be recorded as bleeding and as stroke; note that subarachnoid, subdural, and epidural hemorrhages will be recorded as bleeding events
- *primary ischemic* – stroke without focal collections of intracranial blood; hemorrhagic conversion of a primary ischemic infarction will be recorded
- *uncertain* – no imaging or autopsy data available. Death within 30 days of stroke onset will be considered a fatal stroke.

**3) Transient ischemic attack (TIA)**

An event matching the definition for stroke but lasting less than 24 hours will be considered a transient ischemic attack (TIA). The duration of symptoms for a TIA will be recorded, as will the results of any imaging procedures. However, TIA events with neuroimaging documentation of a cerebral infarction in the appropriate location for the clinical syndrome will be recorded as an ischemic stroke.

**4) Non-central nervous system systemic embolism (SE)**

Non-central nervous system systemic embolism is defined as abrupt vascular insufficiency associated with clinical or radiologic evidence of arterial occlusion in the absence of other likely mechanisms (eg, trauma, atherosclerosis, instrumentation). In the presence of atherosclerotic peripheral vascular disease, diagnosis of embolism to the lower extremities requires angiographic demonstration of abrupt arterial occlusion.

2 – Events on-treatment analysis

| CVA, n (%) | 15 (1.9) |
| --- | --- |
| TIA, n (%) | 7 (0.9) |
| Pulmonary embolism, n (%) | 1 (0.1) |
| DVT, n (%) | 3 (0.4) |
| Myocardial infarction, n (%) | 11 (1.4) |
| Bleeding, n (%) | 80 (10.0) |
| All-cause mortality, n (%) | 71 (8.9) |
| cardiovascular death | 38 |
| non-CV death | 33 |

3 – Bleeding sites

| Gastrointestinal | 23 |
| --- | --- |
| Intracranial | 3 |
| Genitourinary | 29 |
| Musculoskeletal | 5 |
| Skin | 6 |
| Epistaxis | 7 |
| Surgery site | 3 |
| Other | 2 |
| Not reported | 4 |

4 – Baseline characteristics according to dose groups

|  |  | Total | normal^‡^ | reduced^¶^ | p-value |
| --- | --- | --- | --- | --- | --- |
| Number of patients |  | 799 | 690 (86.4) | 109 (14.6) |  |
| Male gender, *n (%)* |  | 489 (61.2) | 426 (61.7) | 63 (57.8) | 0.433 |
| Age, mean ± SD |  | 69.8 ± 11.0 | 68.7 ± 10.4 | 77.0 ± 11.9 | <0.001 |
| Age groups, n (%) |  |  |  |  | <0.001 |
|  | <65 | 228 (28.5) | 214 (31.0) | 14 (12.8) |  |
|  | 65 - 74 | 303 (37.9) | 278 (40.3) | 25 (22.9) |  |
|  | ≥ 75 | 268 (33.5) | 198 (28.7) | 70 (64.2) |  |
| BMI (kg/m2), mean ± SD |  | 27.4 ± 5.0 | 27.5 ± 5.0 | 26.9 ± 5.1 | 0.29 |
| CrCl (ml/min), mean ± SD |  | 65.3 ± 13.7 | 67.1 ± 12.3 | 53.8 ± 16.1 | <0.001 |
| Prior stroke/TIA, n (%) |  | 113 (14.1) | 88 (12.8) | 25 (22.9) | 0.005 |
| Prior PE/DVT, n (%) |  | 30 (3.8) | 21 (3.0) | 9 (8.3) | 0.008 |
| Myocardial infarction, n (%) |  | 102 (12.8) | 80 (11.6) | 22 (20.2) | 0.013 |
| Coronary artery disease, n (%) |  | 170 (21.3) | 136 (19.7) | 34 (31.2) | 0.006 |
| Peripheral artery disease, n (%) |  | 44 (5.5) | 34 (4.9) | 10 (9.2) | 0.071 |
| Congestive heart failure, n (%) |  | 108 (13.5) | 82 (11.9) | 26 (23.9) | 0.001 |
| Hypertension, n (%) |  | 455 (56.9) | 385 (55.8) | 70 (64.2) | 0.099 |
| Diabetes mellitus, n (%) |  | 123 (15.4) | 100 (14.5) | 23 (21.1) | 0.076 |
| Current smoking, n (%)* |  | 89 (11.1) | 77 (11.2) | 12 (11.0) | 0.933 |
| Alcohol abuse, n (%) |  | 127 (15.9) | 110 (15.9) | 17 (15.6) | 0.933 |
| Prior major bleeding, n (%) |  | 23 (2.9) | 17 (2.5) | 6 (5.5) | 0.078 |
| Malignancy n (%) |  | 117 (14.6) | 97 (14.0) | 20 (18.3) | 0.239 |

Note: *data missing of 102 patients. ^†^data missing of 204 patients. ^‡^e.g. dabigatran 150mg, apixaban 5mg, rivaroxaban 20mg, edoxaban 60mg. ^¶^ e.g. dabigatran 110mg, apixaban 2,5mg, rivaroxaban 15mg, edoxaban 30mg.
